# Modeling and design of thin bending wooden bilayers

**DOI:** 10.1371/journal.pone.0205607

**Published:** 2018-10-16

**Authors:** Philippe Grönquist, Falk K. Wittel, Markus Rüggeberg

**Affiliations:** 1 Empa, Applied Wood Materials, Überlandstrasse 129, 8600 Dübendorf, Switzerland; 2 ETH Zurich, Institute for Building Materials, Stefano-Franscini-Platz 3, 8093 Zürich, Switzerland; Oregon State University, UNITED STATES

## Abstract

In recent architectural research, thin wooden bilayer laminates capable of self-actuation in response to humidity changes have been proposed as sustainable, programmed, and fully autonomous elements for facades or roofs for shading and climate regulation. Switches, humidistats, or motor elements represent further promising applications. Proper wood-adapted prediction models for actuation, however, are still missing. Here, a simple model that can predict bending deformation as a function of moisture content change, wood material parameters, and geometry is presented. We consider material anisotropy and moisture-dependency of elastic mechanical parameters. The model is validated using experimental data collected on bilayers made out of European beech wood. Furthermore, we present essential design aspects in view of facilitated industrial applications. Layer thickness, thickness-ratio, and growth ring angle of the wood in single layers are assessed by their effect on curvature, stored elastic energy, and generated axial stress. A sensitivity analysis is conducted to identify primary curvature-impacting model input parameters.

## Introduction

Wood, as a sustainable and natural-grown fiber composite material, has been used over many centuries as mankind’s research on wood and wood-based products has enabled countless applications. More recently, novel and innovative concepts for application as self-actuation-capable, humidity-responsive structures have arisen in applied research for biomimetic architecture [1–8]. Bi-layered wooden structures, capable of complex and extensive shape changes in function of a given geometrical setup and change in ambient climate have been envisaged for diverse uses including shading elements [7], climate-adaptive facades [9], or motor elements [1]. The promising and sustainable principle found its inspirations in nature where anisotropic biological materials with inherent bi-layered and differential fiber structure use humidity changes to generate movement [10–12].

Transferring such innovative concepts to industrial production standards with essential economic benefits requires high reliability of the performance of the elements according to given design and specific application. Next to this, the accurate prediction of actuation as a function of geometry and climate change is crucial. A model was derived by Timoshenko [13] for predicting bending of thin bi-metal strips as a function of temperature change. This concept has recently been applied to the material wood as a linear-elastic prediction of thin bending wooden bilayers [1]. However, the consideration of the essential physical characteristics of the material wood for such simple models is missing. Wood, a biological, anisotropic and hygroscopic material shows a strong moisture-dependency of its material parameters that should be incorporated when modeling its mechanical behavior [14, 15]. Furthermore, as a consequence of orthotropic material behavior, wood should not be treated as isotropic when reduced to two dimensions (2D) for modeling.

We present a simple, wood-adapted, 2D linear-elastic model for predicting curvature for a given moisture content change of thin bending bilayers, based on Refs. [1, 13]. The model is validated using experimental data collected on cross-grained bilayers made out of European beech wood. Using the model, we assess geometrical design aspects such as thicknesses, thickness-ratio, or growth ring angle of the wood in single layers in function of available climate and wood species. These insights help in avoiding potential secondary problems such as adhesive-bond delamination or increased deformation prediction errors. Furthermore, we analyze the effects of these parameters on resulting curvature, stored elastic energy, and maximum axial elastic stresses. Important dimensioning characteristics are suggested in view of optimized structures for industrial application.

High variability in material parameters, a natural characteristic inherent to wood [16], in combination with fabrication tolerances resulting from wood-processing, affecting bilayer geometry, may considerably influence resulting deformation magnitude and thus the precision of the model. To assess these uncertainties, we make use of total Sobol’ indices [17] for a sensitivity analysis on each model input parameter. Through partial variances, the impact of variability in each input parameter on the variability of the model output, i.e., bilayer curvature, can be quantified. Critical parameters can be identified and separated from less important ones in view of efficient in-situ sample measurements and model parameter-updating for facilitated industrial processes.

## Theory

### Elastic properties of European beech

**Fig 1:**
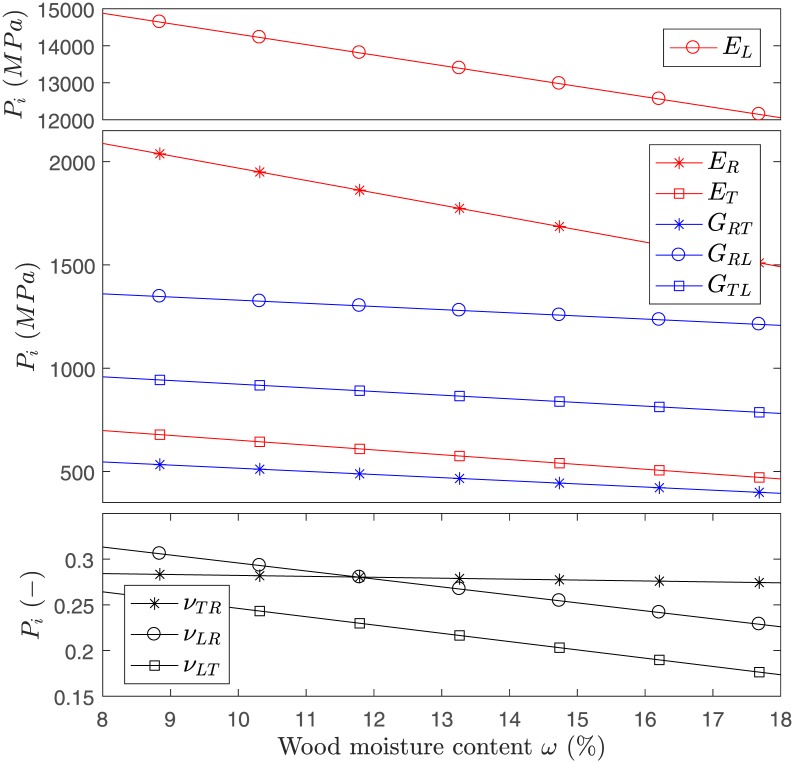
Elastic properties for European beech. Properties *P*_*i*_ as function of moisture *ω* as found in Refs [14] and [15]. The moisture interval [8%, 18%] roughly corresponds to a relative humidity range of [35%, 85%] (adsorption at 85% and desorption at 35%).

Considering Hooke’s law in three dimensional Euclidean space ***σ*** = **C*ε***, a strain-state (***ε*** = *ε*_*kl*_**e**_*k*_ ⊗ **e**_*l*_) is mapped onto a stress state (***σ*** = *σ*_*ij*_**e**_*i*_ ⊗ **e**_*j*_) by a 4^*th*^-order stiffness tensor **C** (**C** = *C*_*ijkl*_**e**_*i*_ ⊗ **e**_*j*_ ⊗ **e**_*k*_ ⊗ **e**_*l*_). The compliance tensor **S** (**S** = **C**^−1^), for an orthotropic material such as wood, is expressed in terms of the engineering constants (w.r.t a basis **e**_1_, **e**_2_, **e**_3_) and in Voigt-notation as

$$\begin{array}{c} S=[\begin{array}{cccccc} E_{1}^{-1} & -\nu_{21}E_{2}^{-1} & -\nu_{31}E_{3}^{-1} & 0 & 0 & 0\\ -\nu_{12}E_{1}^{-1} & E_{2}^{-1} & -\nu_{32}E_{3}^{-1} & 0 & 0 & 0\\ -\nu_{13}E_{1}^{-1} & -\nu_{23}E_{2}^{-1} & E_{3}^{-1} & 0 & 0 & 0\\ 0 & 0 & 0 & G_{23}^{-1} & 0 & 0\\ 0 & 0 & 0 & 0 & G_{31}^{-1} & 0\\ 0 & 0 & 0 & 0 & 0 & G_{12}^{-1} \end{array}] \end{array}$$ (1)

where *E*_*i*_ (Young’s moduli), *G*_*ij*_ (shear moduli), and *ν*_*ij*_ (Poisson’s ratios) can be collected from mechanical characterization experiments. In the case of wood, the local anatomical growth directions *L*, *T*, and *R* (Longitudinal or grain direction, tangential, and radial direction) can be assigned in arbitrary order to the basis **e**_1_, **e**_2_, **e**_3_. Hereby, the representative elementary volume is considered far away from the pith, i.e., the growth-ring-curvature is neglected. Elastic material properties have been collected for the hardwood species European beech (*Fagus sylvatica L*.), in *L*, *R*, and *T* directions as functions of wood moisture content *ω* in Ref [14]. A linear dependence on *ω* is assumed for the species beech as *P*_*i*_ = *b*_0_ + *b*_1_*ω*, where *P*_*i*_ stands for the *i*^*th*^ property *P* that represent the engineering constants, and *b*_0_ and *b*_1_ are coefficients found in Ref [15]. Fig 1 shows *P*_*i*_ in terms of *ω* as they are used in the present study.

### 2D linear elastic model for wooden bilayer curvature

**Fig 2:**
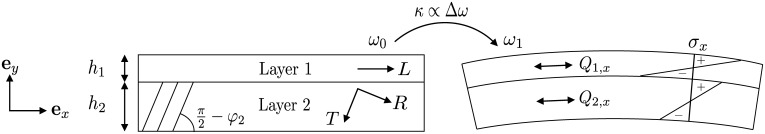
2D schematic of wooden bilayer. Initial flat (wet, *ω*_0_) and resulting deformed state (dry, *ω*_1_) after drying (*ω*_0_ > *ω*_1_). Global 2D coordinate system (basis **e**_*x*_, **e**_*y*_) and local anatomical directions (*R*, *T*, *L*) for layers 1 and 2 with growth ring inclination *φ*_2_ of layer 2. Passive layer (layer 1) with thickness *h*_1_ and active layer (layer 2) with thickness *h*_2_. Stiffnesses *Q*_*i*,*x*_ in **e**_*x*_-direction of layer *i* and axial stress distribution *σ*_*x*_ over cross-section.

The 3D elastic material law ***ε*** = **S*σ*** with **S** as described in Eq (1) is hereafter reduced to 2D by assuming (i) a plane strain state, and alternatively, (ii) a plane stress state in. A 2D schematic of a wooden bilayer is represented in Fig 2, where the undeformed state (with initial wood moisture content *ω*_0_) is shown along a deformed state (curvature *κ*, wood moisture content *ω*_1_ < *ω*_0_, for drying). Typically, the wood is oriented such that in layer 1, *L* aligns with **e**_*x*_ and in layer 2 *R* aligns with **e**_*x*_ and *T* with **e**_*y*_. This results in a stiff and resisting layer 1 (called “passive” layer, very low swelling in *L* direction) to block axial deformation at the interface (an adhesive bond) coming from the driving layer 2 (the “active” layer, high swelling and shrinkage in *R*- or *T*-direction). For the resulting curvature *κ* of a 2D model, the stiffnesses *Q*_*i*,*x*_ in direction of **e**_*x*_ of each layer (*i* = 1, 2) are of interest. For cases (i) and (ii), and using the same basis as for Eq 1, they are derived as

$$\begin{array}{c} Q_{i,x}^{\left(i\right)}=\frac{E_{1}}{1-\nu_{13}\nu_{31}},\;\;Q_{i,x}^{\left(ii\right)}=\frac{E_{1}-\nu_{12}^{2}E_{2}}{1-\nu_{12}\nu_{21}}, \end{array}$$ (2)

assuming a uni-axial stress state ***σ***^(2*D*)^ = (*σ*_*x*_, 0, 0)^*T*^ to act on the bilayer.

The curvature for a cross-grained wooden bilayer structure is a function of the applied difference in moisture content. Existing models assume a direct proportionality *κ* ∝ Δ*ω* [1]. The presumed proportionality factor (referred to as *K*) is, in the case of wood, not constant but is itself a function of moisture (*K* = *K*(*ω*)). For any bilayer configuration, *K* contains the effects of geometry and material parameters and is obtained by formulating equilibrium, compatibility, and a deformation equilibrium at the interface of two bonded Euler-Bernoulli beams [13]. In terms of Eq 2 and considering the basis **e**_*x*_,**e**_*y*_ in Fig 2, the term is derived as

$$\begin{array}{c} K=[\frac{h_{1}+h_{2}}{2}+\frac{2(Q_{1,x}I_{1}+Q_{2,x}I_{2})}{h_{1}+h_{2}}\left(\frac{1}{Q_{1,x}h_{1}}+\frac{1}{Q_{2,x}h_{2}}\right){]}^{-1}\left(\alpha_{2,x}-\alpha_{1,x}\right) \end{array}$$ (3)

where $I_{1}=h_{1}^{3}/12$ and $I_{2}=h_{2}^{3}/12$ are the second moments of area per unit width of bilayer-strip. *h*_1_ and *h*_2_ denote layer thicknesses and *α*_1,*x*_ and *α*_2,*x*_ are the differential swelling coefficients in bilayer axial direction (**e**_*x*_) such that *α* = *ε*^*ω*^Δ*ω*^−1^ where *ε*^*ω*^ is the swelling strain. For beech wood, typical values used are *α*_*L*_ = 0.0001, *α*_*R*_ = 0.0019, and *α*_*T*_ = 0.0040 in %^−1^ [15]. Here, we assume *α* as remaining constant over Ω, otherwise, the swelling strain would read *ε*^*ω*^ = ∫_Ω_ *αdω*. Ω stands for a moisture domain such that for beech wood Ω ⊂ [0, *FSP*(%)] contains all possible states of *ω* where *FSP* denotes the fiber saturation point. For any moisture increment Δ*ω* ∈ Ω, the change in layer-thickness (direction **e**_*y*_) of layer *i* can thus be calculated as Δ*h*_*i*_ = *h*_*i*_*α*_*i*,*y*_Δ*ω*, and added (swelling) or subtracted (shrinkage) to the actual layer thicknesses. Next to updating thicknesses and mechanical parameters over changes of *ω* ∈ Ω, the growth ring inclination *φ*, considered only in layer 2 as *φ*_2_ (Fig 2) affecting the orientation of the local wood-*RT*-plane, results in a rotation of the properties (analogous to an element-rotation in Mohr’s circle). In layer 2, *α* is thus transformed as *α*_2,*x*_ = *α*_2,11_ cos^2^(*φ*_2_) + *α*_2,22_ sin^2^(*φ*_2_), and equally, the transformed stiffness, where *Q*_2,11_ is calculated as in Eq 2, reads

$$\begin{array}{c} Q_{2,x}=Q_{2,11}\cos^{4}\varphi_{2}+Q_{2,22}\sin^{4}\varphi_{2}+2\left(Q_{2,12}+2Q_{2,66}\right)\sin^{2}\varphi_{2}\cos^{2}\varphi_{2}. \end{array}$$ (4)

Considering all moisture dependencies, thickness changes of layers and growth ring inclination, the resulting curvature *κ* over any Ω is calculated as

$$\begin{array}{c} \kappa =\int_{\Omega }Kd\omega +\kappa_{0}. \end{array}$$ (5)

The total work performed, or resilient elastic energy stored, *W* ([*W*] = *Nm* ⋅ *m*^−2^), to achieve *κ* over Ω can be expressed, in the 2D case, as

$$\begin{array}{c} W=\frac{1}{2}\int_{\Omega }K^{2}Fd\omega +W_{0},\;\;F_{i}=\frac{4{\left(Q_{i}I_{i}+Q_{i}I_{i}\right)}^{2}}{Q_{i}h_{i}{\left(h_{1}+h_{2}\right)}^{2}}+Q_{i}I_{i}. \end{array}$$ (6)

The factor *F*_*i*_ stands for contribution from layer *i*. For a bilayer with passive layer 1 and active layer 2, *F* = *F*_1_ + *F*_2_.

If, as in the calculation of Eq 3, it is assumed that the neutral axes situate at half the thicknesses of each layer and that stiffnesses *Q*_*i*_ are constant over cross-section, the axial maximal and absolute stress values in layer *i* at layer-interface and layer-edge (in **e**_*x*_-direction) can be expressed by

$$\begin{array}{c} \sigma_{i}=\int_{\Omega }K\sqrt{\frac{2F_{i}Q_{i}}{h_{i}}}d\omega +\sigma_{i,0}. \end{array}$$ (7)

We note that *h*, *I*, *Q*, *K*, *W*, *F*, and *σ* are all functions of *ω*, and that in a strict sense, the integrals of Eqs 5–7 can be evaluated for any given Δ*ω* ∈ Ω. However, for practical application, it is convenient to replace them using an incremental approach. For Eq 5, e.g. $\kappa -\kappa_{0}\approx \sum_{i}^{n}\Delta K_{i}\Delta \omega_{i}$ for Δ*ω*_*i*_ = Ω/*n*, where in each increment, the geometry and engineering constants (all *E*, *G*, and *ν*) are updated for the actual moisture level.

## Materials and methods

### Bilayer validation samples

Six different configurations of cross-grained bilayers were fabricated from beech wood (Fagus sylvatica L.) grown in the region of Zurich, Switzerland. Three samples were fabricated for each configuration. The wood was initially conditioned at a wet climate of 85% r.h. (relative humidity) and 20°*C*. The single layers were cut as strips of 120 mm length and 20 mm width. For the six configurations, the thicknesses were varied according to the ratio *r* = *h*_1_(*h*_1_ + *h*_2_)^−1^ as shown in Table 1 where *h*_1_ and *h*_2_ denote thickness of layers 1 and 2 respectively (Fig 2). A growth ring angle *φ*_2_ of zero was chosen for all experimental validation samples. The layers were glued using a one-component polyurethane glue (1cPUR, Purbond HB S709, Henkel & Cie AG, Switzerland) and manufacturer’s standards were applied for the gluing procedure. The samples were placed inside a 35% r.h. and 20°*C* climate room to dry and curvatures were measured after *t* = 1, 2, 4, 6, 8, 24, and 48 hours using image analysis (polynomial fits to edge-thresholds). Simultaneously, wood moisture contents *ω*_*t*_ at time *t* were measured using a gravimetric determination method, i.e. $\omega_{t}=\left(m_{t}-m_{0}\right)m_{0}^{-1}$, where *m*_*t*_ is the sample mass at time *t* and *m*_0_ is the oven-dry sample mass. Finally, experimental data is verified against Eq 5.

**Table 1:** Configurations of experimental sample-set. Layer thicknesses *h*_1_ and *h*_2_ of passive layers (layer 1) and of active layers (layer 2) in *mm* along ratios *r*.

*h*_1_ (*mm*)	0.6	0.8	1.2	2.2	3.2	3.9
*h*_2_ (*mm*)	4.1	4.1	4.1	4.1	4.1	4.1
*r* = *h*_1_(*h*_1_ + *h*_2_)^−1^	0.12	0.17	0.22	0.34	0.43	0.49

### Sensitivity analysis

Defining Eq 5 as the model with uncertain input, where each parameter is following a specific probability density function (PDF) with given parameters or moments, model output variability can be assessed using a Monte-Carlo (MC) sampling approach. This procedure allows for calculating partial variances of the model parameters and their combinations w.r.t. the model output variance. We make use of total Sobol’ indices to conduct a global-type sensitivity analysis of the model [17, 18]. The indices, normalized values between 0 and 1, for each parameter *i*, equal the contribution of variability of that parameter to the model output variability.

For the analysis, the *Matlab*-based uncertainty quantification tool *UQLab* was used [19]. The engineering constants *E*_*L*_, *E*_*R*_, and *E*_*T*_ entering the model (for the plane strain case), the three swelling coefficients *α*_*L*_, *α*_*R*_, and *α*_*T*_, the two thicknesses *h*_1_, and *h*_2_, and the growth ring orientation *φ*_2_, were sampled using 10^7^ MC samples. We assume a log-normal PDF for the material properties ($\{E_{L},E_{R},E_{T},\alpha_{L},\alpha_{R},\alpha_{T}\}∼LN$), where a coefficient of variation (*COV* = *σ*/*μ*) of 10% is attributed to each property, and a Gaussian PDF for the geometrical properties ($\{h_{1},h_{2},\varphi_{2}\}∼N$) where standard deviations (*σ*) of 0.1 *mm*, 0.1 *mm* and 1° are attributed respectively. Exemplary, four beech bilayer configurations were analyzed (described along Results). The mean values *μ*_*i*_ (first moments of the input PDFs of *i*) for the material properties are taken as in Fig 1. The described input parameters were chosen in view of direct applicability in industry. The Young’s moduli, the differential swelling coefficients, and the geometry are deemed easier to record than other input parameters (i.e. shear moduli and Poisson ratios).

## Results

### Model validation with experimental data

**Fig 3:**
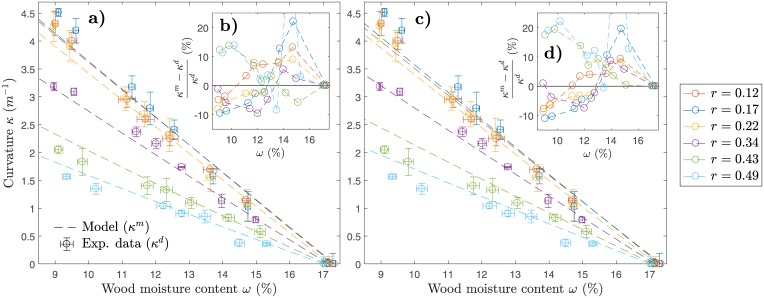
Model curvature (*κ*^*m*^) vs. experimental validation data (*κ*^*d*^). a) Plane strain model versus data (error-bars denote positive and negative standard deviations); b) Error in prediction of plane strain model with respect to experimental data ((*κ*^*m*^ − *κ*^*d*^)(*κ*^*d*^)^−1^); c) Plane stress model versus data; d) Error in prediction of plane stress model with respect to experimental data.

The plane strain (i) and the plane stress (ii) model formulations were validated against the obtained experimental results. Fig 3 presents the data of measured (*κ*^*d*^) and predicted (*κ*^*m*^) curvature versus wood moisture content (*ω*). The measured curvatures exhibit significant dependence on the ratio *r*. A maximum of *κ*^*d*^ ≈ 4.5 *m*^−1^ was reached for *r* = 0.17 and a minimum of *κ*^*d*^ ≈ 1.5 *m*^−1^ was reached for *r* = 0.49 after 48 hours in 35% r.h. climate, at *ω* ≈ 9.5%. For the cases (i) and (ii), errors in prediction are displayed starting with a value of zero at initial conditions (*ω* ≈ 17.5%). It can be seen, that the prediction errors are lower for model (i) than for model (ii). In fact, for low values of *r* (*r* = 0.12 and *r* = 0.17), both models slightly under-predict *κ*^*d*^, while for higher values (*r* = 0.43 and *r* = 0.49), *κ*^*d*^ is over-predicted. At a value of *r* = 0.34, which corresponds approximately to *h*_1_: *h*_2_ = 1: 2, both models predict *κ*^*d*^ very well ($R_{i}^{2}=0.986$ and $R_{ii}^{2}=0.992$) with an absolute prediction error lower than 10%. Over all configurations, model (i) reached a mean goodness of fit (*R*^2^) to experimental data of ${\bar{x}}_{R_{i}^{2}}=0.972$ and model (ii) of ${\bar{x}}_{R_{ii}^{2}}=0.950$.

### Parametric study on curvature, elastic energy, and axial stress

**Fig 4:**
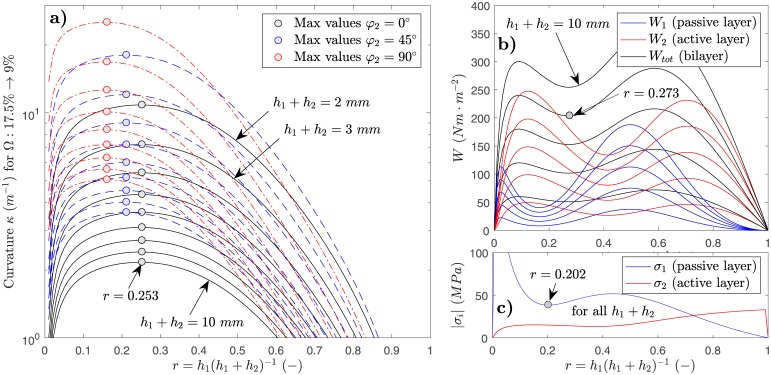
Parametric study using plane strain model. a) Curvatures *κ* (semi-log plot) versus all possible thickness ratios *r* over an exemplary moisture domain of Ω: 17.5% → 9% for beech bilayers (*L*//***e***_***x***_ in layer 1 and *R*//***e***_***x***_ in layer 2, *T* in other directions). Black lines *φ*_2_ = 0°, blue lines *φ*_2_ = 45°, and red lines *φ*_2_ = 80°. *h*_1_ + *h*_2_ is chosen from 2 *mm* (top lines) to 10 *mm* (bottom lines) in steps of 1 *mm*. b) Strain energy *W* vs. *r* for same setup with *φ*_2_ = 0°. Blue and red lines represent contribution to *W*_*tot*_ (black line) from passive and active layers. *h*_1_ + *h*_2_ is chosen from 2 *mm* (bottom lines) to 10 *mm* (top lines) in steps of 2 *mm* c) Absolute values of axial stresses at interface *σ*_*i*_ for layer *i*, two curves represent all possible *h*_1_ + *h*_2_.

In this section, *κ*, *W*, and *σ*_*i*_ are analyzed as function of *r* (from 0 to 1), of bilayer-thickness *h*_1_ + *h*_2_ (from 2 to 10 *mm*), and of *φ*_2_ (0°, 45°, and 90°). A complete range of feasible configurations of thin beech bilayers is thus represented. The plane strain model for a moisture variation of Ω: 17.5% → 9% was used, and the results are displayed in Fig 4a (configurations described in figure caption). Highest curvatures are reached at a same value of *r* regardless of *h*_1_ + *h*_2_. This optimal value of *r* (maximizing *κ*, e.g. at *r* = 0.253 for *φ*_2_ = 0°) is, however, shifted in function of *φ*_2_. The higher *φ*_2_ is chosen, the higher *κ* for a same value of *h*_1_ + *h*_2_. High curvatures of *κ* ≈ 10 − 25 *m*^−1^ result for 2 *mm* thick bilayers (depending on *φ*_2_). By choosing higher values of *h*_1_ + *h*_2_, *κ* drastically drops in magnitude and seems to converge to a minimum value (at a given *r*) for large *h*_1_ + *h*_2_ according to the spacing of the curves in the semi-log plot for *κ*.

It can be seen, that *W*_*tot*_ is a linear function of total thickness *h*_1_ + *h*_2_ for any *r* (Fig 4b). Considering the contribution of layer 1 (*W*_1_) and layer 2 (*W*_2_) to *W*_*tot*_ separately, it is seen that there are values of *r* for each layer i where *W*_*i*_ is either minimized or maximized. As for *κ*, these *r* remain independent of *h*_1_ + *h*_2_. *W*_1_ is minimized where *W*_2_ is maximized and vice versa, and *W*_*tot*_ is minimized at *r* = 0.273. Surprisingly, the axial interface-stress does not depend on total bilayer thickness *h*_1_ + *h*_2_ but only on *r* (Fig 4c). Stresses in the passive layer are found to show higher dependence on *r* as those of the active layer. The minimum stress of the passive layer situates at *r* = 0.202, whereas for the active layer, stresses seem to remain approximately constant between *r* = 0.1 and *r* = 0.4.

### Model input parameter sensitivity analysis

**Fig 5:**
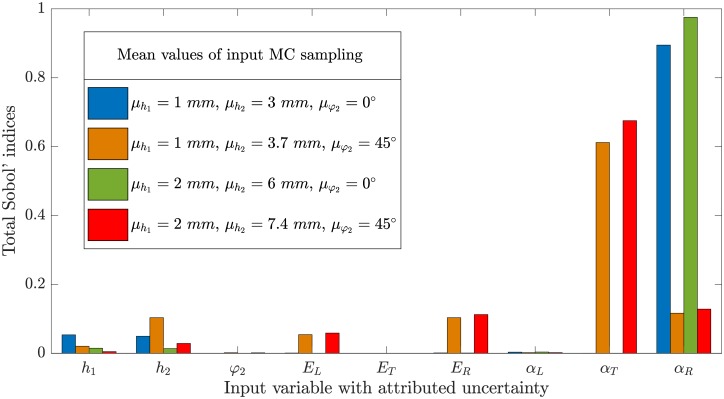
Computed total Sobol’ indices using plane strain model. Four different exemplary configurations are considered. Two different thicknesses of passive layer are considered: *h*_1_ = 1 *mm* and *h*_2_ = 2 *mm*. Thicknesses of active layers follow optimal values for ratio *r* (see in Fig 4a) for respective cases *φ*_2_ = 0° (*r* = 0.253) and *φ*_2_ = 45° (*r* = 0.212). These parameters constitute the first moments of the respective pdfs (*μ*_*i*_).

Results of the sensitivity analysis are displayed in Fig 5. Four different model configurations were considered for which *r* was set as the optimal value maximizing *κ* according to Fig 4 and Ω: 17.5% → 9%. In the case of $\mu_{h_{1}}=1$ *mm* and $\mu_{\varphi_{2}}=0^{{}^{\circ}}$, most of the model response variability can be attributed to *α*_*R*_. Input variabilities in thicknesses *h*_1_ and *h*_2_ also contribute to a small amount. In the case of $\mu_{h_{1}}=2$ *mm*, the contribution from the thicknesses is marginal, and all model variability seems to be exclusively attributed to the variability of *α*_*R*_. Setting $\mu_{\varphi_{2}}=45^{{}^{\circ}}$, a major shift from *α*_*R*_ to *α*_*T*_ in total Sobol’ indices can be observed. When $\mu_{\varphi_{2}}=45^{{}^{\circ}}$, additionally, some contribution seems to be attributed to Young’s moduli in bilayer axial direction **e**_**x**_ of the passive layer (*E*_*L*_) and the active layer (*E*_*R*_, but surprisingly not *E*_*T*_). Regardless of the model configuration, no effects of input variability of the parameters *φ*_2_, *E*_*T*_, and *α*_*L*_ are visible.

## Discussion

### Model validity

It is seen that the proposed 2D linear elastic model can predict curvature of beam-like bilayer structures with narrow widths and thin layers. Model precision tends to get worse when high or low values of *r* (close to 1 or 0) are chosen. A plane strain state assumption predicted data better than a plane stress state assumption. This is in line with the known phenomena of strong out-of-plane effects of anisotropic materials when the out-of-plane material axis is stronger than the in-plane axis, as in the case of the active layer in a wooden bilayer. Besides, we note that curvature in such a bilayer is not a uni-axial phenomenon but, will happen about two perpendicular axes simultaneously. A wooden bilayer plate will, however, tend to minimize the change in Gaussian curvature. Curvature out of plane is thus expected to affect axial curvature to some extent. Besides modeling only a reduced geometry (2D), known beech-wood-inherent deformation mechanisms such as visco-elasticity [20, 21], mechano-sorption [15], and plasticity [22] are here neglected for the sake of simplicity. Also, the presented model assumes steady-state moisture conditions, which is an ideal assumption, supposedly valid for thin layers. Time and moisture-diffusion effects, especially for increased layer thicknesses, are certainly of further interest when understanding wooden bilayer mechanical behavior. Overall, the relation *κ* ∝ Δ*ω* appears to hold true despite the highly non-linear nature of the model and the complex physical effects in experimental data.

### Design principles

Given the validity of the presented model, design aspects can be discussed using parametric studies as presented in Fig 4. For any arbitrary configuration defined by local orientations *L*, *T*, and *R*, material properties, and growth ring angle *φ*_2_, the optimal thickness ratio *r*_*opt*_, for which the curvature *κ* is maximized (over any Ω) can be calculated applying *dκ*/*dr* = 0 and solving for *r*. In Ref [23] an exemplary solution for the case of isotropic bimaterials with constant material properties is given as $E_{1}h_{1}^{2}=E_{2}h_{2}^{2}$, where *E*_1_ and *E*_2_ are the layer stiffnesses. For the presented model and wood, the optimal ratio *r*_*opt*_ is, however, dependent on many input parameters. It was seen that *κ* can be maximized, or *W*_*tot*_ and *σ*_*i*_ can be minimized, and that in either case *r*_*opt*_ is different. We state that in certain range of *r*, however, there is a tendency for the maximum curvature being reached while simultaneously, the system minimizes stored elastic energy and axial stresses. Furthermore, model prediction of validation data was better in the case of the samples with *r* close to *r*_*opt*_, tending to the conclusion that maximized *κ* and minimized *W* and *σ* influence model precision and justify the use of a 2D linear elastic model, as presented, to predict *κ* for such cases. Given the opportunity to vary *h*_1_ + *h*_2_ or Δ*ω* in order to set a target *κ*, we thus recommend to always set *r* to an optimal value *r*_*opt*_ when designing wooden bilayers.

### Increasing model precision

The model parameters of relevance were identified as the swelling coefficients in the active layer in all of the cases studied. Nearly all variability in *κ* was attributed to the variability of *α*_*R*_ (or *α*_*T*_). Other parameters appear to be of much lower relevance (vanishing total Sobol’ indices), and it can be concluded that measuring and updating the model with those parameters is not necessary. By measuring the swelling coefficients and updating the model input to *in situ* values, most of the epistemic uncertainty in the model response can be reduced to nearly only including the aleatory uncertainty of that input (given the measurement is accurate). Aleatory uncertainty in model input parameters, however, can never be reduced. For wood, as a naturally grown material with high variability, a precision range of predicted model response compared to experimental validation data can realistically not be lower than the range of spread in measured input material parameters.

### Remarks on cracking and delamination

Under mechanical, but mainly moisture loading, cross-laminated wooden structures are prone to cracking [24]. For thin wooden bilayers, two basic failure modes can be considered: Axial delamination of the adhesive bond at the layer interface, and transverse cracking in the active layer. Both modes supposedly originate from a combination of the crack separation modes I and II (mode-mixity). At the bilayer edges, the zero axial stress boundary condition results in a stress intensity factor *K*_*I*_ at the interface. In combination with the interface-inherent stress intensity factor *K*_*II*_ due to the shear transfer, the edge-near interface can be identified as a zone with high risk of failure. We refer to the work of Refs [25, 26] where the failure of multilayers is analyzed, and, as general finding, the term $h_{i}\sigma_{i}^{2}Q_{i}^{-1}\Gamma_{c}^{-1}$ is to be minimized to reduce failure risk. Γ_*c*_ denotes the interface toughness and is a configuration-specific parameter to be experimentally determined. The dominant influence of the axial stress *σ*_*i*_ on the term can directly be recognized, underlining the importance of choosing a ratio *r*, for which axial stress is minimized (for beech bilayers with *φ*_2_ = 0, *r*_*opt*,*σ*_ ≈ 0.2). Layer thickness *h*_*i*_, stiffness *Q*_*i*_, and Γ_*c*_, only have secondary effects on failure risk. We state that failure was not observed for the experimental bilayer samples in this study, but that it can hypothetically occur for very high Δ*ω* in combination with unsuitable design.

## Conclusions

We derive and propose a simple 2D linear elastic model for predicting curvature of thin wooden bilayers as a function of moisture change. The model takes into account moisture-dependent orthotropic properties for wood and a physically accurate reduction to two dimensions. Experimental curvature data of beech bilayers is accurately modeled within a 10% precision range given an optimal ratio of layer thicknesses. In the design process, anatomical orientations of wood in each layer, the wood species, the growth ring angle in the active layer, the applied moisture difference, and the bilayer thickness combined with the thickness ratio of passive and active layers need to be considered. An optimal thickness ratio can be found for every configuration where either bilayer curvature is maximized or the passive layer axial stress at the interface and the edge is minimized. Axial stresses are found independent of total bilayer thickness and only depend on thickness ratio and applied moisture content difference. Further, they exert a considerable influence on delamination risk. The variability in axial swelling coefficient in the active layer was identified to have a decisive impact on bilayer curvature. Finally, we recommend considering effects such as moisture gradients or time-dependent mechanical behavior for modeling and design of wooden bilayers with increased layer thickness.
